# Supplementary material for: What will the cardiovascular disease slowdown cost? Modelling the impact of CVD trends on dementia, disability, and economic costs in England and Wales from 2020–2029
Source: PLoS One. 2022 Jun 29;17(6):e0268766. doi: 10.1371/journal.pone.0268766 (PMC9242440; doi:10.1371/journal.pone.0268766)
Supplement: S1 File — (DOCX) [file pone.0268766.s001.docx]

Technical Appendices

**What will the cardiovascular disease slowdown cost? Modelling the impact of CVD trends on dementia, disability, and economic costs in England and Wales from 2020-2029.**

Brendan Collins^1*^, Piotr Bandosz^2^, Maria Guzman-Castillo^3^, Jonathan Pearson-Stuttard^4^, George Stoye^5^, Jeremy McCauley^6^, Sara Ahmadi-Abhari^4^, Marzieh Araghi^4^, Martin J Shipley^7^, Simon Capewell^1^, Eric French^8^, Eric J Brunner^7^, Martin O’Flaherty^1^

Appendices

1. Overview of IMPACT Better Aging Model and Matrix calculations
2. Summary of Methods for Attaching Costs and QALYs to the IMPACT BAM Model
3. Sensitivity analyses varying value of QALY and discount rates

# Appendix 1. Overview of IMPACT Better Aging Model

(Note this section mainly reproduced from Guzman-Castillo et al., 2017 supplemental appendix, with tables and figure named as in the original appendix. <https://www.thelancet.com/journals/lanpub/article/PIIS2468-2667(17)30091-9/fulltext> )

The IMPACT Better Ageing Model (IMPACT-BAM) is a discrete-time Markov model which follows the progression of a healthy population (aged 35+ years old) from England and Wales into ten different health states characterised by the presence or absence of cardiovascular disease (CVD), cognitive impairment and functional impairment from 2006 to 2025. The model structure is presented in Figure S. 7, the health states are described in Table S. 6 and transition probabilities, pi,j, in Table S. 7

Prior to simulation, we populated each state in the model based on ONS population estimates in 2006 (start year) and prevalence of the above conditions from ELSA, except for the new cohort of 35-year olds that enters the system through the disease-free state (see section 5.10). The simulation allows individuals to move to other states in the model. The arrows in Figure S. 7 indicate the possible movements of people between these ten states, which are governed by one-year probabilities of transition.

For example, a healthy 55-year man starts the simulation in state 1 (Disease-free state) in 2006. He moves to state 2 (CVD) in 2007 after having a stroke. In 2008 he could either die from complications of the stroke (he moves to state 9), any other causes (he moves to state 10) or he could develop cognitive impairment (moving to state 3) or disability (moving to state 5). As above, movements to any state are driven by transition probabilities. Detailed information on the estimation of transition probabilities is provided in sections 5.2 to 5.9

Then, to calculate the number of people with disability in year t, we count the number of people in the states that represent disability (states 5, 6, 7 and 8) at year t. To calculate the number of individuals with dementia, we count the number of people in state 6 and 7 at year t, and do so similarly for other conditions. These numbers are then used to calculate the prevalence of any disease in the model. Detailed information on output calculation can be found in section 7.

Figure S. 7: IMPACT-BAM model structure. Transitions to death states 9 and 10 are possible from any state.
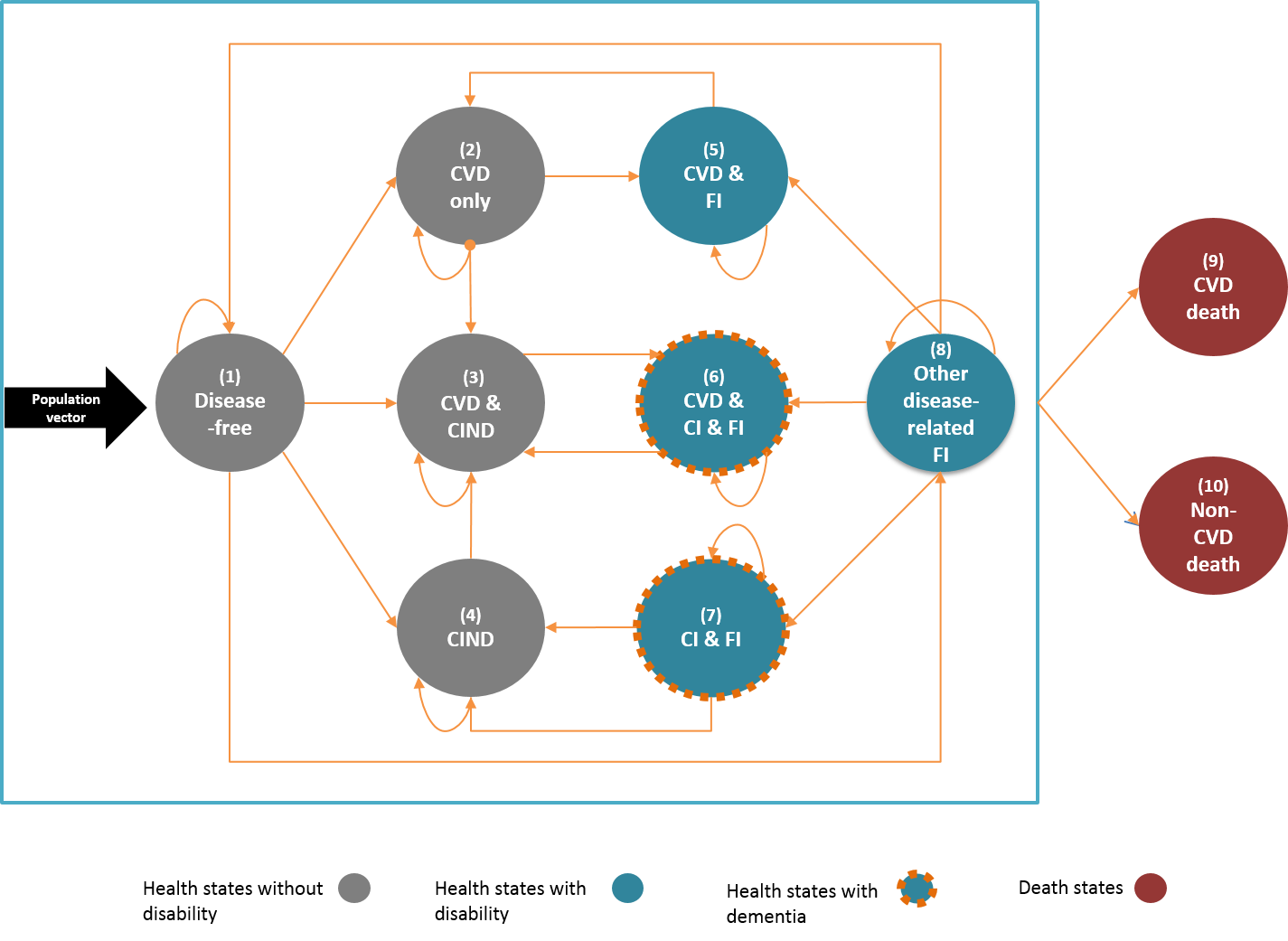


Table S. 6: Description of the health states

| Health state | Name | Description |
| --- | --- | --- |
| 1 | Disease-free population | People free of cardiovascular disease (CVD), cognitive impairment (CI) or functional impairment (FI) |
| 2 | CVD only | Cardiovascular disease |
| 3 | CVD and CIND | Cardiovascular disease and cognitive impairment no dementia |
| 4 | CIND | Cognitive impairment no dementia |
| 5 | CVD and FI | Cardiovascular disease and functional impairment |
| 6 | CVD, CIND and FI | Cardiovascular disease and dementia (cognitive + functional impairment) |
| 7 | DEMENTIA | cognitive + functional impairment |
| 8 | Other disease-related FI | Functional impairment no related to CVD or/and Dementia |
| 9 | CVD death | Death from CVD causes |
| 10 | Non-CVD death | Death from a different cause than CVD |

Table S. 7: Description of transition probabilities. Each transition probability is stratified by sex and age

| 1-year transition probability | From | To |
| --- | --- | --- |
| $p_{1,1}$ | Disease-free population | Disease-free population |
| $p_{1,2}$ | Disease-free population | CVD only |
| $p_{1,3}$ | Disease-free population | CVD and CIND |
| $p_{1,4}$ | Disease-free population | CIND |
| $p_{1,8}$ | Disease-free population | Non-CVD/Non-dementia FI |
| $p_{1,9}$ | Disease-free population | CVD death |
| $p_{1,10}$ | Disease-free population | Non-CVD death |
| $p_{2,2}$ | CVD only | CVD only |
| $p_{2,3}$ | CVD only | CVD and CIND |
| $p_{2,5}$ | CVD only | CVD and FI |
| $p_{2,9}$ | CVD only | CVD death |
| $p_{2,10}$ | CVD only | Non-CVD death |
| $p_{3,3}$ | CVD and CIND | CVD and CIND |
| $p_{3,6}$ | CVD and CIND | CVD and Dementia |
| $p_{3,9}$ | CVD and CIND | CVD death |
| $p_{3,10}$ | CVD and CIND | Non-CVD death |
| $p_{4,4}$ | CIND | CIND |
| $p_{4,7}$ | CIND | Dementia |
| $p_{4,9}$ | CIND | CVD death |
| $p_{4,10}$ | CIND | Non-CVD death |
| $p_{5,5}$ | CVD and FI | CVD and FI |
| $p_{5,2}$ | CVD and FI | CVD only |
| $p_{5,9}$ | CVD and FI | CVD death |
| $p_{5,10}$ | CVD and FI | Non-CVD death |
| $p_{6,6}$ | CVD and dementia | CVD and dementia |
| $p_{6,3}$ | CVD and dementia | CVD and CIND |
| $p_{6,9}$ | CVD and dementia | CVD death |
| $p_{6,10}$ | CVD and dementia | Non-CVD death |
| $p_{7,7}$ | Dementia | Dementia |
| $p_{7,4}$ | Dementia | CIND |
| $p_{7,9}$ | Dementia | CVD death |
| $p_{7,10}$ | Dementia | Non-CVD death |
| $p_{8,1}$ | Other disease-related FI | Disease-Free population |
| $p_{8,5}$ | Other disease-related FI | CVD and FI |
| $p_{8,6}$ | Other disease-related FI | CVD and Dementia |
| $p_{8,7}$ | Other disease-related FI | Dementia |
| $p_{8,8}$ | Other disease-related FI | Non-CVD/Non-dementia FI |
| $p_{8,9}$ | Other disease-related FI | CVD death |
| $p_{8,10}$ | Other disease-related FI | Non-CVD death |

# Inputs and Calculations

## Case definitions

Cardiovascular disease (represented in the model by states 2,3,5 and 6 in Figure S.7) was defined as having a diagnosis of cardiovascular disease, myocardial infarction, stroke and/or angina; equivalent to the ICD10 codes I00-I99, G45, Q200-Q289, M300-M319, D180-D189, A182, K550-K559, R00-R009, R071-R074, R098, R230, R590-R599 and R943.

Cognitive impairment no dementia (states 3 and 4) was defined as impairment in two or more domains of cognitive function (such as orientation to time, immediate and delayed memory, verbal fluency, and numeracy function), or a score higher than 3.6 on the Informant Questionnaire for Cognitive Decline (IQCODE)10 administered for subjects who were unable to participate in the study.^6^

Functional impairment (states, 5, 6, 7 and 8)was defined as the inability to independently perform one or more activities of daily living (ADL). The ADLs included getting in or out of bed, walking across a room, bathing or showering, using the toilet, dressing, cutting food and eating. We distinguished four disability states: state 5 defined as CVD-related disability, state 7 as dementia-related disability, state 6 as CVD and dementia related disability and state 8 as other disease-related disability defined as other forms of disability not linked to CVD or dementia. To quantify the burden of CVD- related disability, we did not consider the contributions of state 6 (CVD and dementia related disability) as we wanted to isolate the disability burden associated to CVD only. Similarly for dementia-related disability.

Dementia (states 6 and 7) was defined based on the co-existence of cognitive impairment and functional impairment or a report of a doctor diagnosis of dementia by the participant or carer.

## Incidence of CVD (P_1, 2_, P_4, 3,_ P_8,5_)

Denote $P\left( CVD \right)$to be the incidence of CVD. To calculate CVD incidence, $P\left( CVD \right),$we obtained 2-year incidence rates from the English Longitudinal Study of Ageing (ELSA) and fitted a logistic regression model of the form:

$logit incidence=\beta_{0}+\beta_{age}{age}_{35}+\beta_{sex}sex+\beta(sex*{age}_{35})$+$\beta_{state}state$

Where ${age}_{35}$is individual age centred at 35 and *state* are those states (states 1, 4 and 8) from where transitions to CVD states (states 2, 3 and 5) are allowed.

From the logistic regression estimates, 2-year transition probabilities were computed which were later transformed into gender specific 1-year transition probabilities for single years of age.

In our model, we defined states 2 and 4 (CVD-only and CIND-only) as mutually exclusive (i.e. a patient who is in the CVD-only state does not have CIND at the same time and vice versa). Therefore, to calculate the transition probability $p_{1,2}$we subtract the proportion of patients who have both CVD and CIND, $p_{1,3}$

$p_{1,2}=P\left( CVD \right)-p_{1,3}$

## Incidence of CIND (P_1, 4_, P_2, 3,_ P_8,7_)

Denote $P\left( CIND \right)$to be the incidence of “cognitive impairment no dementia”. To calculate CIND incidence, $P\left( CIND \right),$2-year incidence rates from ELSA were modelled as follows:

We fitted a logistic regression model of the form:

$logit incidence=\beta_{0}+\beta_{age}{age}_{50}+\beta_{sex}sex+\beta\left( sex*{age}_{50} \right)+\beta\left( sex*{age}_{50}^{2} \right)+\beta_{state}state$

Where ${age}_{50}$is individual age centred at 50 and *state* are those states (states 1, 2 and 8) from where transitions to CI states (states 4, 3 and 7) are allowed

This allowed us to compute 2-year transition probabilities that were later transformed into gender specific 1-year transition probabilities for single years of age. The incidence rates from ELSA are likely to be underestimated due to higher drop out of those who do develop cognitive impairment.

In our model, we defined states 2 and 4 (CVD-only and CIND-only) as mutually exclusive (i.e. a patient who is in the CVD-only state does not have CIND at the same time and vice versa). Therefore, to calculate the transition probability $p_{1,4}$, we subtract the proportion of patients who have both CVD and CIND, $p_{1,3}$

$p_{1,4}=P\left( CIND \right)-p_{1,3}$

## Incidence of CVD and CIND (P_1, 3_, P_8,6_)

We assume that CVD and CIND are independent events. Therefore, ($p_{1,3}=P(CVD \cap CIND))$from a healthy state, $p_{1,3}=P(CVD)\times P(CIND)$ from above formula

Similarly for $p_{8,6}$

## Incidence of functional impairment states (P_1, 8_, P_2, 5,_ P_3,6,_ P_4,7_ )

We obtained the 2-year incidence rates for functional impairment ELSA and fitted logistic regression models of the form:

${logit incidence FI=\beta}_{0}{+\beta}_{age}{age}_{35}+\beta_{sex}sex++\beta_{state}state+\beta(sex*{age}_{35})+\beta(state*{age}_{35})$

Where ${age}_{35}$is individual age centred at 35 and *state* are those states (states 1, 2, 3 and 4) from where transitions to FI states (states 8, 5, 6 and 7) are allowed.

This allowed 2-year transition probabilities to be computed which were later transformed into gender specific1-year transition probabilities for single years of age. These transition probabilities do not have a calendar effect.

## Recovery from functional impairment states (P_8, 1_, P_5, 2_, P_6,3_ and P_7,4_)

We obtained the 2-year incidence rates for functional impairment ELSA and fitted logistic regression models of the form:

${logit incidence FI=\beta}_{0}{+\beta}_{age}{age}_{35}+\beta_{sex}sex++\beta_{state}state+\beta(sex*{age}_{35})+\beta(state*{age}_{35})$

Where ${age}_{35}$is individual age centred at 35 and *state* are those FI states (states 8, 5, 6 and 7) from where transitions to states without FI (states 1, 2, 3 and 4) are allowed.

This allowed 2-year transition probabilities to be computed which were later transformed into gender specific1-year transition probabilities for single years of age. These transition probabilities do not have a calendar effect.

## Transition probabilities from state i to the death states (P_i, 9,_ P_i, 10_)

The computation of the transition probabilities $p_{i,9}$involved three steps:

For the first step, CVD mortality probabilities of CVD up 2025 in 5-year age bands were calculated using the Bayesian Age Period Cohort (BAPC) model,^7^ with ONS mortality and population estimates from 1982-2012 for England and Wales as inputs.

The curve fitting tool in MATLAB was then used to obtain CVD mortality probabilities for single years of age, starting at 35 years old. The probabilities are estimated using piecewise cubic Hermit interpolation to estimate values that lie between known data points, with the monotonicity and the shape of the data preserved. We denote these probabilities of death by ${m\_cvd}_{a,t}$, where, *a* is the age of individual and *t* the calendar year.

For the second step, we calculated mortality rates from ELSA for the age groups 50-59, 60-69, 70-79 and 80-89 and fitted two logistic regression models of the form:

$logit cvd\_death=\beta_{0}+\beta_{age}{age}_{35}+\beta_{male}male+\beta(male*{age}_{35})$

$logit cvd\_death=\beta_{0}+\beta_{age}{age}_{35}+\beta_{male}male+\beta(male*{age}_{35})$+$\boldsymbol{\beta}_{\mathbf{s}}\mathbf{state}$

Where ${age}_{35}$is individual age centred at 35, $\boldsymbol{\beta}_{\mathbf{s}}$ is a vector containing the *β* coefficients for all the states.

The first equation allowed us to compute gender specific baseline transition probabilities for single years of age. We defined these as $\tilde{p}_{0,9,a}$

The second equation allowed us to compute gender and state-specific transition probabilities for single years of age. We defined these as $\tilde{p}_{i,9,a}$

To estimate how different the state-specific transition probabilities are from the baseline transition probabilities we calculated${cvd}_{a,i}=\frac{\tilde{p}_{i,9,a}}{\tilde{p}_{0,9,a}}$.

The probabilities of death, ${m\_cvd}_{a,t}$, are the probabilities of dying (from CVD) regardless of the state an individual is coming from, similar to the baseline transition probabilities $\tilde{p}_{0,9,a}$from the ELSA study. The ${m\_cvd}_{a,t}$ are calculated using the entire England and Wales population and allow for cohort and calendar effects and are preferred over the $\tilde{p}_{0,9,a}$.

To allow for each subject’s initial state, the ${m\_cvd}_{a,t}$ were multiplied by the factor ${cvd}_{a,i}$ to obtain the age, gender and state-specific transition probabilities$p_{i,9,a}$.

Transition probabilities $p_{i,10,a}$were calculated in the same manner.

## Calendar effect for CVD and CIND incidence

Let$\Delta_{a,t+1}=\frac{{m\_cvd}_{a,t+1}}{{m\_cvd}_{a,t}}$ where ${m\_cvd}_{a,t}$ is the age-specific probability of death from CVD causes in year *t*. Therefore, $\Delta_{a,t+1}$is an age-specific adjustment factor describing how different the probability of CVD death in year *t+1* is from the probability of CVD death in the previous year *t*.

We assume that annual changes in CVD incidence mirror the annual changes in CVD mortality as observed in ELSA (See Figure S. 8). In other words, we assume the annual percentage change in CVD incidence equals to the annual percentage change in CVD mortality. Therefore, to obtain the incidence of CVD allowing for a calendar effect, we multiplied ${P(CVD)}_{a,t+1}$by$\left( \Delta_{a,t+1} \right)$.

Likewise, we assume that these annual changes in CVD incidence would also affect$p_{8,5}$, thus the same calendar was applied.

However, the incidence of CIND, ${P(CIND)}_{a,t+1},$is assumed to decrease by 2.7% per calendar year, ie, ${P(CIND)}_{a,t+1}$ = 0.973 * ${P(CIND)}_{a,t}.$

The above annual decline for CIND was estimated with data collected over 6 waves of ELSA (2002-2013) and using an elaborate model that takes into account losses to follow-up and mortality. The results of these analyses suggested that the calendar trend per year is -2.7 (95% confidence interval -2.9, -2.4) %.^8^

Likewise, we assume an annual 2.7% decrease for $p_{8,6}$ and $p_{8,7}$.The calculations of $p_{1,2},p_{1,3}$ and $p_{1,4}$, proceed as previously described.

Figure S. 8: Age and sex standardised cardiovascular incidence and mortality rates in the English Longitudinal Study of Ageing 2002-2013


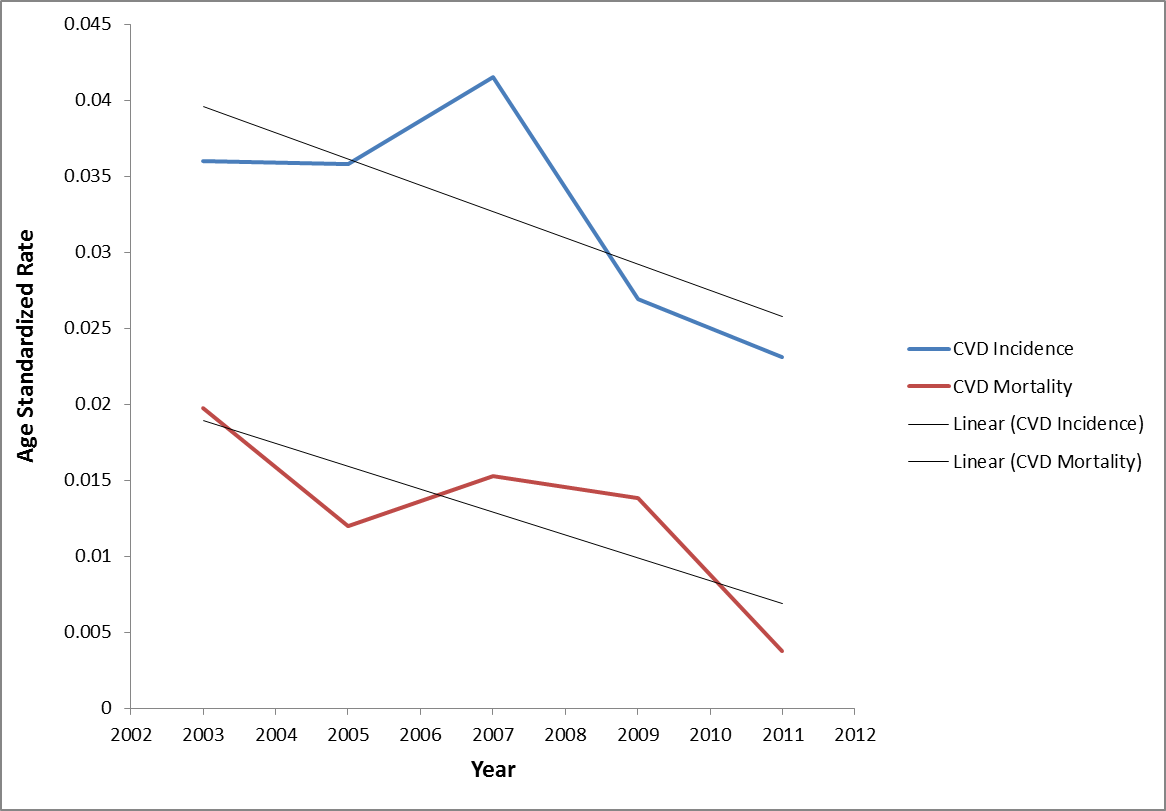


## Recurrent state transition probabilities

The recurrent state transition probabilities such as p_1,1,_ p_2,2_, p_3,3,_ etc. were calculated using the following formula:

$p_{i,i}=1-\sum_{j=1}^{J} p_{i,j}$, where *J* is a vector containing the states (other than *i* itself) to where a transition from state i is possible.

## Prevalence of initial states

We obtained the 2-year prevalence rates for states 2, 3, 4, 5, 6, 7 and 8 from ELSA for 5-year age groups. Due to the small number, it was assumed that those aged <50 have a prevalence probability of cognitive impairment equal to zero. This was done by dividing the number of people in each state by the total number of individuals in that age-sex strata in the pooled ELSA data and attributed to 2006 which is the mid-point of the ELSA data collection period (2002-2013).

We then used the curve fitting tool in MATLAB to obtain data for single year of age starting at 35 years old.

ELSA contains information on 48 individuals aged 34 to 36. Forty-seven (98%) of these individuals were free of CVD, cognitive impairment, dementia and disability. Therefore, we assumed that the new cohort of 35s entering the model at each year is free of disease. This assumption has no effect on the outputs reported in the manuscript, as the 35-year-olds entering the model in 2006 (beginning of the simulation) will be 54 years old by 2025, while the outputs reported in this study are for those 65 and older.

# Matrix calculations

The following table contains the steps to calculate the Markov model

Table S. 8: Matrix notation for programming purposes mainly. (Example for men)

| Matrix formulation (Example for men) | Description |
| --- | --- |
| $\boldsymbol{p\_m}_{\mathbf{a}}=\left[ {p\_m}_{a,1},{p\_m}_{a,2},\ldots,{p\_m}_{a,10} \right]$ | Column vector containing prevalence rates for all states for men aged *a* |
| $M_{a,0}$ | Scalar containing initial population men aged *a* |
| $\mathrm{Tm}_{a,t}=\left[ \begin{matrix} p_{1,1,a,t} & \cdots& p_{1,10,a,t} \\ \vdots& \ddots& \vdots\\ p_{10,1,a,t} & \cdots& p_{10,10,a,t} \end{matrix} \right]$ | Matrix for men aged *a*, containing the transition probabilities |
| $\mathbf{m}_{\mathbf{a,t}}=\left[ m_{a,t,1},m_{a,t,2},\ldots,m_{a,t,10} \right]$ | Column vector containing the number of men aged *a* in each state at time *t* |
| For $t=0$  $\mathbf{m}_{\mathbf{a},\mathbf{0}}=M_{a,0}\cdot{\mathbf{p}\_\mathbf{m}}_{\mathbf{a}}$ | |
| For $t=n$  $\mathbf{m}_{\mathbf{a},\mathbf{t}}=\mathbf{m}_{\mathbf{a}-\mathbf{1},\mathbf{t}-\mathbf{1}}\cdot\left[ T_{a-1} \right]^{T}$  $\mathbf{m}_{\mathbf{a,t}}\mathbf{=}\left[ \begin{matrix} m_{a-1,t-1}p_{1,1,a-1,t-1}+m_{a-1-1,t-1}p_{2,1,a-1,t-1}+\ldots+m_{a-1,t-1}p_{10,1,a-1,t-1}, \\ m_{a-1,t-1}p_{1,2,a-1,t-1}+m_{a-1,t-1}p_{2,2,a-1,t-1}+\ldots+m_{a-1,t-1}p_{10,2,a-1,t-1,} \\ \boldsymbol{\ldots,} \\ m_{a-1,t-1}p_{1,10,a-1,t-1}+m_{a-1,t-1}p_{2,10,a-1,t-1}+\ldots+m_{a-1,t-1}p_{10,10,a-1,t-1} \end{matrix} \right]$ | |

# Appendix 2. Summary of Methods for Attaching Costs and QALYs to IMPACT Better Aging Model

# 1 Costs

All costs were inflated to 2018/19 prices using the GDP deflator, October 2018. The costs for the model were produced by single year of age (from age 35 to 100 inclusive), gender, and IMPACT BAM Markov model state (out of 8 model states). This yields a maximum of 1,056 separate cost values representing each possible age/gender/state combination. However, not all costs were disaggregated by gender, and some were produced by age groups instead of single year of age. Figure 1 shows the number of synthetic people in the model by health state and age.

Figure 1. Number of people in IMPACT BAM state by single year of age, 2018.

CVD = cardiovascular disease. CI=cognitive impairment. FI = functional impairment.

## 1.1 Healthcare Costs

For healthcare costs we have used Hospital Episode Statistics (HES) data that has been matched with ELSA wave 7 (collected in 2014/15) and matched based on Healthcare Resource Group (HRG) national tariffs. These tariffs are prices (paid by commissioners to hospitals for the care they provide) rather than costs. However, they are based on NHS Reference Cost data and so represent the average costs of healthcare activity. The data separately covers inpatient, outpatient and A&E activity. We used the regression-based values rather than the actual averages in order to impute costs for people who are not present in the original dataset, (i.e. if a combination of age group/gender/IMPACT BAM health state has a population of zero in ELSA). Ordinary Least Squares regression estimates are shown in Table 1.

Table 1. OLS regression results for hospital costs (3), ELSA wave 7 hospital costs (by type), age bands.

|  | Hospital costs (£, 2018/19 prices) | | | |
| --- | --- | --- | --- | --- |
|  | Total | Inpatient | Outpatient | A&E |
| Constants | 490.3 | 297.4 | 146.1 | 46.81 |
| Health state |  |  |  |  |
| CVD | 774.1 (98.8) *** | 587.9*** | 138.1*** | 48.12*** |
|  | (98.80) | (87.83) | (16.35) | (6.860) |
| CVD+CI | 1025*** | 727.7*** | 130.8*** | 166.5*** |
|  | (252.1) | (224.2) | (41.72) | (17.51) |
| CI | 245.5* | 167.8 | 55.09** | 22.65** |
|  | (139.1) | (123.7) | (23.02) | (9.658) |
| CVD+FI | 1657*** | 1211*** | 346.8*** | 98.76*** |
|  | (133.9) | (119.1) | (22.16) | (9.300) |
| CVD+CI+FI (CVD+Dementia) | 2156*** | 1788*** | 210.4*** | 157.3*** |
|  | (220.2) | (195.7) | (36.43) | (15.29) |
| CI+FI (Dementia) | 1621*** | 1402*** | 122.6*** | 96.20*** |
|  | (196.5) | (174.7) | (32.51) | (13.64) |
| FI without CVD or dementia | 1121*** | 910.1*** | 171.5*** | 39.11*** |
|  | (87.81) | (78.06) | (14.53) | (6.097) |
| Sex |  |  |  |  |
| Female | -12.23 | -15.41 | 1.470 | 1.709 |
|  | (53.24) | (47.33) | (8.809) | (3.696) |
| Age group |  |  |  |  |
| 55-59 | -98.97 | -101.8 | 0.376 | 2.408 |
|  | (123.5) | (109.8) | (20.44) | (8.578) |
| 60-64 | 24.19 | 19.01 | 12.11 | -6.938 |
|  | (116.4) | (103.4) | (19.25) | (8.079) |
| 65-69 | 227.7** | 173.3* | 59.67*** | -5.199 |
|  | (115.8) | (103.0) | (19.16) | (8.041) |
| 70-74 | 384.3*** | 316.6*** | 70.20*** | -2.508 |
|  | (121.5) | (108.0) | (20.11) | (8.438) |
| 75-79 | 666.7*** | 525.7*** | 127.9*** | 13.02 |
|  | (124.5) | (110.7) | (20.60) | (8.646) |
| 80-84 | 632.1*** | 492.5*** | 116.8*** | 22.74** |
|  | (140.6) | (125.0) | (23.26) | (9.761) |
| 85+ | 536.6*** | 480.0*** | 35.60 | 20.95** |
|  | (151.3) | (134.5) | (25.03) | (10.50) |
| Observations | 7980 | 7980 | 7980 | 7980 |
| R-squared | 0.080 | 0.066 | 0.072 | 0.057 |

**Example, interpreting Table 1**

If someone is in CI health state, male, aged 62; total hospital costs = (490.3 (constant) +245.5(CI)+0(male, not shown)+24.19(age 60-64))=£760. This would then be multiplied by 3.04 to calibrate to total healthcare costs (excluding prescribing costs) giving a value of £2,310 [Actual value is £2313 due to rounding differences]. With prescribing costs of £148 this would give total healthcare costs of £2461.

We multiplied the HES-based costs (by age/gender/and IMPACT BAM state) by a factor of 3.04 to be consistent with OBR estimates of total healthcare costs by age (projection for year 2020/21 from 2016 OBR Fiscal Risks Report, deflated to 2019 prices).(1) Prescribing costs, which are consistent with other estimates, were then added. The total healthcare costs per person by single year of age look similar both to the OBR costs (as would be expected given they have been calibrated to the OBR costs), and also to estimates from Asaria (2018) (2). There are many other estimates of costs and utility for dementia and for CVD, and for CHD and stroke more specifically.

Figure 2. Estimated average healthcare cost per person-year in all health states by age, England and Wales, 2018/19 prices; comparison of IMPACT BAM, OBR, and Asaria estimates.

### Estimated Cost of Prescriptions Dispensed in the Community

Community prescribing costs were added on to the healthcare costs described above. We used data from ELSA wave 6 on the details of up to 40 prescribed medications currently being taken by each respondent. For each prescription, the data lists the British National Formulary (BNF) paragraph, section, and chapter number. This is matched to the Net Ingredient Costs (NICs) contained in Prescription Cost Analysis compiled by Health and Social Care Information Centre.(3) NIC is the basic cost of a drug as used in primary care. This is the cost at list price excluding VAT, (i.e. the price listed in the national Drug Tariff or in standard price lists). The NIC is not necessarily the price the NHS paid as it does not factor any contract prices or discounts, dispensing costs, fees or prescription charge income. The average number of prescribed medicines in the ELSA data is only 3.36 (see Table 3), which is much lower than the average number of items prescribed per head of the UK population, which was 18.7 in 2012 (see Table 2). Part of the explanation for this is that ELSA only records the prescribed medications currently being taken by each respondent at the time of the survey, and it also does not consider how often they need a repeat prescription of that medication. We adjust the average number of medications in ELSA to match the national average and then sum the NIC for all medications for each person. This gives an overall average annual cost of prescriptions dispensed in the community of £189, and a total cost of £6.3billion. As with healthcare costs, we used the regression-based estimates in the model. Because some respondents in ELSA have zero prescriptions, we used a two-part regression; a probit for the presence of prescription medicines, and OLS for the cost of medicines consumed (Table 7). The final estimates are a combination of the probit and OLS estimates.

Table 2. Prescriptions Dispensed in the Community - England, 2007 to 2017

| Year | Items | NIC (£) | Average NIC per prescription item (£) | Average number of items per head of population | NIC per head of population (£) | England population (millions) |  |
| --- | --- | --- | --- | --- | --- | --- | --- |
| 2007 | 796,298,021 | 8,372,667,329 | 10.51 | 15.5 | 162.95 | 51.4 |  |
| 2008 | 842,502,224 | 8,325,492,244 | 9.88 | 16.3 | 160.67 | 51.8 |  |
| 2009 | 885,999,283 | 8,539,421,190 | 9.64 | 17.0 | 163.60 | 52.2 |  |
| 2010 | 926,657,637 | 8,834,379,813 | 9.53 | 17.6 | 167.82 | 52.6 |  |
| 2011 | 961,528,633 | 8,805,067,360 | 9.16 | 18.1 | 165.80 | 53.1 |  |
| 2012 | 1,000,503,693 | 8,523,134,718 | 8.52 | 18.7 | 159.33 | 53.5 |  |
| 2013 | 1,030,084,459 | 8,625,091,941 | 8.37 | 19.1 | 160.12 | 53.9 |  |
| 2014 | 1,064,587,183 | 8,852,687,094 | 8.32 | 19.6 | 162.98 | 54.3 |  |
| 2015 | 1,083,676,422 | 9,266,676,092 | 8.55 | 19.8 | 169.14 | 54.8 |  |
| 2016 | 1,104,131,794 | 9,204,891,304 | 8.34 | 20.0 | 166.55 | 55.3 |  |
| Table Source: Table 1 of “Prescriptions Dispensed in the Community - England, 2007 to 2017”  Publication Date: 28 June 2018  Author: Prescribing and Medicine Team, NHS Digital  Data Source: PCA based on British National Formulary (BNF) September 2014  Costs are in nominal £s. | | | | | | | |

Table 3. Prescriptions Costs (£) per person-year by health state in ELSA Wave 6

| **Table:** Prescriptions Costs in ELSA Wave 6 | | | | | | | |  |  |
| --- | --- | --- | --- | --- | --- | --- | --- | --- | --- |
| State | Number of Prescriptions (unadjusted) | Number of Items (adjusted) |  | Average NIC per  item (£) |  | Average Annual Cost  (unadjusted, £) | Average Annual Cost  (adjusted, £) | Rep. Population 2018 (millions) | Total cost  (millions, £) |
|  |  |  |  |  |  |  |  |  |  |
| Overall | 3.36 | 18.70 |  | 10.09 |  | 33.95 | 189.11 | 33.12 | 6,262.62 |
|  |  |  |  |  |  |  |  |  |  |
| Free | 2.35 | 13.06 |  | 10.47 |  | 24.94 | 138.90 | 25.91 | 3,598.94 |
| CVD | 6.43 | 35.84 |  | 7.20 |  | 50.43 | 280.89 | 1.82 | 510.97 |
| CVD+CI | 5.89 | 32.83 |  | 9.26 |  | 71.04 | 395.69 | 0.40 | 158.57 |
| CI | 3.08 | 17.18 |  | 10.95 |  | 31.84 | 177.37 | 1.50 | 265.46 |
| CVD+FI | 8.48 | 47.24 |  | 9.76 |  | 86.49 | 481.74 | 0.70 | 335.89 |
| CVD+CI+FI (CVD+Dementia) | 8.68 | 48.34 |  | 10.89 |  | 92.87 | 517.30 | 0.16 | 83.35 |
| CI+FI (Dementia) | 5.32 | 29.63 |  | 12.17 |  | 66.93 | 372.80 | 0.55 | 205.11 |
| FI without CVD or dementia | 5.44 | 30.30 |  | 10.97 |  | 58.68 | 326.83 | 2.08 | 679.91 |
| Data from ELSA wave 6 (2012). Drug costs estimated by matching BNF codes in ELSA with NIC data from the PCA (2012).  Number of prescriptions are adjusted so that the average number of items in a year is 18.7. | | | | | | | | | |

Figure 3. Community prescribing cost per year (£2019) by IMPACT BAM health state and age; average of males and females.

Figure 4. Total healthcare costs(£2019) including prescribing, by IMPACT BAM health state and age; average of males and females.

##

## 1.2 Social Care Costs

ELSA asks questions regarding social care resource use (e.g. hours used in the last week) in waves 6 and 7. We attached costs to the hours of social care used data, based on PSSRU reference costs per hour, inflated to 2018/19 costs by multiplying by 1.0375 (see Table 4, 5, 6). One of the social care hours used questions asks about cleaners, however we could not find a cost estimate for a cleaner and therefore have used the national living wage +50% for this. As with healthcare costs, we used the regression-based estimates in the model. In the same way as with prescription medicines, because a lot of respondents in ELSA report zero hours of social care use, we used a two-part regression; a probit for the presence of social care use, and OLS for the amount of resource use for people who use it (Table 7). For daycare we only had receipt of daycare, not hours, and therefore only used a probit model and applied average annual cost from PSSRU reference costs (£7,280 in 2016/17 costs). Overall, the social care costs add up to around £7.8billion (based on multiplying resource use by population in health states from 2018). The total costs of daycare across the population is estimated to be around £1.0billion. The estimated social care and daycare totals show the type of pattern that might be expected, rising significantly after age 85 and being highest for people with CVD and dementia.

Table 4. Social care unit costs used for resources in ELSA.

| Resource | Unit cost per hour (£2018/19) | Original Unit cost (£) | Source | Year | Page | Notes |
| --- | --- | --- | --- | --- | --- | --- |
| Cleaner | 11.95 | 11.745 | Based on national living wage (£7.83) +50% to account for additional costs and travel time. | 2018 |  |  |
| Care/nursing home staff | 4.71 | 4.56 | PSSRU Reference Costs  https://www.pssru.ac.uk/pub/uc/uc2017/services.pdf | 2016/17 | 33 | Table 1.1. Based on £762 per week / 168 hours per week |
| Other formal help | 16.10 | 15.52 | PSSRU Reference Costs  https://www.pssru.ac.uk/pub/uc/uc2017/services.pdf | 2016/17 | 37 | Assume same as home care |
| Local authority provided Home care worker/ home help | 26.58 | 25.62 | PSSRU Reference Costs  https://www.pssru.ac.uk/pub/uc/uc2017/services.pdf | 2016/17 | 37 |  |
| Non-Local Authority Home care worker/ home help | 16.10 | 15.52 | PSSRU Reference Costs  https://www.pssru.ac.uk/pub/uc/uc2017/services.pdf | 2016/17 | 37 |  |
|  |  |  |  |  |  |  |
| Daycare | 145.25 (per week) | 140 (per week) | PSSRU Reference Costs  https://www.pssru.ac.uk/pub/uc/uc2017/services.pdf | 2016/17 | 37 |  |
| Residential Care | 39,869 (per year) | 39,156 (per year) | PSSRU Reference Costs <https://www.pssru.ac.uk/pub/uc/uc2018/services.pdf> | 2017/18 | 27-28 | Average of Table 1.1 and Table 1.2 |

Table 5. Usage of Formal Care by IMPACT BAM health state.

| State | No. Obs. |  | Any Help (%) |  | Any Formal Help (%) |  | LA Provided Formal Help (%) |  | Use Day Care (%) |
| --- | --- | --- | --- | --- | --- | --- | --- | --- | --- |
| Free of All | 8009 |  | 7.98% |  | 1.61% |  | 0.10% |  | 0.12% |
| CVD | 1031 |  | 23.79% |  | 4.95% |  | 0.48% |  | 0.51% |
| CVD+CI | 51 |  | 45.10% |  | 5.88% |  | 0.00% |  | 1.96% |
| CI | 204 |  | 24.02% |  | 5.88% |  | 3.92% |  | 0.50% |
| CVD+FI | 380 |  | 79.74% |  | 22.89% |  | 9.21% |  | 2.37% |
| CVD+CI+FI | 138 |  | 71.01% |  | 33.33% |  | 15.94% |  | 7.97% |
| CI+FI (Dementia) | 167 |  | 70.06% |  | 25.75% |  | 14.97% |  | 11.98% |
| FI without CI or CVD | 900 |  | 61.67% |  | 15.00% |  | 4.33% |  | 1.56% |

Table 6. Example of hours per week of care, with unit costs, from ELSA data.

|  | Any Formal Care | | | |  | LA Provided Formal Care | Non-LA Formal Care |
| --- | --- | --- | --- | --- | --- | --- | --- |
| State | Total Home care worker/ home help | Cleaner | Care/ | Other formal help |  | Home care worker/ home help | Home care worker/ home help |
|  |  |  | nursing home staff |  |  |  |  |
| Free of all | 0.02 | 0.02 | 0.01 | 0.05 |  | 0.01 | 0.01 |
| CVD | 0.04 | 0.04 | 0 | 0.06 |  | 0.03 | 0.01 |
| CVD+CI | 0.07 | 0.06 | 0 | 0.02 |  | 0 | 0.07 |
| CI | 0.03 | 0.06 | 0 | 0.53 |  | 0.03 | 0 |
| CVD+FI | 1.11 | 0.18 | 0.01 | 1.07 |  | 0.69 | 0.42 |
| CVD+CI+FI | 3.14 | 0.03 | 1.31 | 1.18 |  | 1.79 | 1.35 |
| CI+FI (Dementia) | 1.43 | 0.07 | 1.38 | 0.28 |  | 1.23 | 0.2 |
| FI without CI or CVD | 1 | 0.13 | 0.15 | 0.16 |  | 0.54 | 0.46 |

Table 7. Regression output using Two-part models (Probit + OLS regression) for social care and daycare

|  | Social Care Total (Home Help + Nursing Staff + Cleaning + Other) - excluding Day Care and Residential | | Day Care |  |  | Social Care Total (Home Help + Nursing Staff + Cleaning + Other) - excluding Day Care and residential | | Day Care |
| --- | --- | --- | --- | --- | --- | --- | --- | --- |
|  | Probit | OLS | Probit |  | Health state | probit | OLS | probit |
| Sex |  |  |  |  | estimates continued from left panel | | | |
| Male | -0.310*** | 2126.2** | -0.121 |  | CVD | 0.335*** | -1230.9 | 0.193 |
|  | (0.0382) | (1073.9) | (0.0814) |  |  | (0.0613) | (1875.8) | (0.166) |
| Age group |  |  |  |  | CVD+CI | 0.462*** | -400.5 | 0.813*** |
| 55-59 | 0.0882 | 3713.6 | 0 |  |  | (0.147) | (3733.5) | (0.240) |
|  | (0.121) | (4437.2) | (.) |  | CI | 0.172 | 1722.6 | 0.660*** |
| 60-64 | 0.188 | 1938.0 | 0 |  |  | (0.105) | (3116.7) | (0.180) |
|  | (0.115) | (4208.7) | (.) |  | CVD+FI | 1.070*** | 6292.9*** | 0.781*** |
| 65-69 | 0.234** | -35.51 | 3.508 |  |  | (0.0625) | (1587.8) | (0.144) |
|  | (0.113) | (4145.1) | (120.9) |  | CVD+CI+FI (CVD+Dementia) | 1.243*** | 9374.2*** | 1.376*** |
| 70-74 | 0.375*** | -1156.3 | 3.753 |  |  | (0.0863) | (1910.9) | (0.145) |
|  | (0.113) | (4087.9) | (120.9) |  | CI+FI (Dementia) | 1.042*** | 8827.8*** | 1.457*** |
| 75-79 | 0.748*** | -1750.8 | 3.787 |  |  | (0.0827) | (1891.7) | (0.137) |
|  | (0.109) | (3927.4) | (120.9) |  | FI without CVD or dementia | 0.929*** | 4053.9*** | 0.788*** |
| 80-84 | 0.914*** | -1035.9 | 3.910 |  |  | (0.0486) | (1404.9) | (0.120) |
|  | (0.112) | (3950.9) | (120.9) |  | Constant | -2.478*** | 3405.2 | -6.565 |
| 85+ | 1.409*** | -529.0 | 4.033 |  |  | (0.102) | (3851.1) | (120.9) |
|  | (0.110) | (3867.5) | (120.9) |  | Observations | 19,863 | 19,863 | 13,001 |
|  |  |  |  |  |  |  |  |  |

Notes: Data from ELSA waves 6 and 7. Reference groups are: Female, Age 35-54, Free of all. Heteroskedasticity robust standard errors are displayed in parentheses. * p < 0.1, ** < 0.05, *** p < 0.01.

Interpreting the social care and daycare regression equations:

| Average Social Care Costs = Normal(Probit Coefficient,0,1)*(OLS regression coefficients) | | | | |
| --- | --- | --- | --- | --- |
|  |  | |  |  |
| IMPACT BAM state | | Fraction Positive from Probit | Annual Cost if Cost>0 (£) | Avg Annual Cost (£) |
| Female, free of all, aged <55 | | 0 | 3,405.2 | 0 |
| Female, CI+FI(Dementia), 75-80 | | 0.2457 | 10,482.2 | 2,576 |

Average Daycare Costs = Normal(Probit Coefficient,0,1)*Annual Unit Cost

| IMPACT BAM state | Fraction Positive from Probit | Annual Cost if Cost>0 (£) | Avg Annual Cost (£) |
| --- | --- | --- | --- |
| Female, free of all, aged <55 | 0.0000 | 7,280 | 0 |
| Female, CI+FI(Dementia), 75-80 | 0.0933 | 7,280 | 679 |

### Residential Care

The need for residential care was estimated using ELSA data using a probit regression. We then assumed an average yearly cost of living in institutional care of £39,156 (based on a 50:50 split between residential and nursing beds, taking the average of the two costs (£34,268 + £44,044)/2), using PSSRU reference costs for 2018, inflated to £39,869 in 2018/19 prices. People in ELSA who reported residential care reported zero hours of all other forms of care which meant there was no double counting in the data. Residential care totals were summed with other social care estimates to get an estimate of total social care costs for each combination of gender, age and health state (Figure 5).

Table 8. Regression output using Probit model for institutional care

|  | Institutional Care  Probit |
| --- | --- |
| Sex |  |
| Male | -0.278 |
|  | (0.0998) |
| Female | 0 |
|  |  |
| Age group | |
| 35-54 | 0 |
|  |  |
| 55-59 | 3.270 |
|  | (112.2) |
| 60-64 | 3.611 |
|  | (112.2) |
| 65-69 | 3.440 |
|  | (112.2) |
| 70-74 | 3.514 |
|  | (112.2) |
| 75-79 | 3.799 |
|  | (112.2) |
| 80-84 | 4.092 |
|  | (112.2) |
| 85-100 | 4.501 |
|  | (112.2) |
| Health state | |
| Free | 0 |
|  |  |
| CVD | 0.0513 |
|  | (0.339) |
| CVD+CI | 0.628 |
|  | (0.398) |
| CI | 0.577 |
|  | (0.298) |
| CVD+FI | 1.008 |
|  | (0.210) |
| CVD+CI+FI (CVD+Dementia) | 2.378 |
|  | (0.186) |
| CI+FI (Dementia) | 2.001 |
|  | (0.187) |
| FI without CVD or dementia | 0.834 |
|  | (0.198) |
|  |  |
| Constant | -7.107 |
|  | (112.2) |
|  |  |
| Obs | 19863 |

Figure 5. Estimated social care spend per person year by health state and age, IMPACT BAM model for England & Wales, 2018/19 prices, average of males and females. Includes day care and residential care.

## 1.3 Opportunity costs of Informal (Unpaid) Care

There is no real consensus on how to include costs of informal (unpaid) care in economic evaluations. Ways to cost informal care may include using average hourly wages of all industries, hourly wages in the formal care sector, or contingent valuation of getting respite from informal care.

There are different types of informal care: active care such as bathing and helping people get dressed, and passive care like sitting with someone, or being ‘on call’ for when active care is required. The UK office for National Statistics (ONS) uses four categories of informal care for its Household Satellite Accounts which are based on Family Resources Survey data:

1. continuous care, where individuals require a continuous carer for a maximum 168 hours per week;
2. practical care, where care is not continuous but involves physical help with paper work, financial matters or other practical help such as shopping, laundry, housework, gardening, doing odd-jobs, taking out for a walk, keeping an eye on someone
3. personal care is classified as help with personal care such as dressing, bathing, washing, shaving, feeding, using the toilet, physical help such as walking, getting up and down the stairs getting into and out of bed, and other sorts of personal help such as preparing meals, giving medicines and change dressings
4. personal / practical which is a mix of the two categories in the same hour.

Overall, based on ONS estimates of the total GVA for unpaid care in the UK for 2016 (£59.45bn after deducting housing inputs)(4), combined with the average hours per recipient of 3,857 per year from 2014, this gives an average cost per hour of informal care of £7.34 in 2016 prices, or £7.76 in 2019 prices (Table 9).

ELSA asks questions regarding informal care. Specifically, respondents are asked about the number of hours of help received in last week from up to 25 different people, ranging from spouses to neighbours. Respondents can either say they received no help or choose from 8 ranges of hours (e.g. “Less than one hour”, “1-4 hours” etc.), with the maximum being “100 hours or more”. To calculate the total number of hours each individual received in a week we took the mid-point of each range (e.g. “1-4 hours” as 2.5 hours) and treated the response “100 hours or more” as 100 hours. We then summed across the 25 different people that each respondent may have received help from. The results can be seen in Table 10. To calculate a yearly total we multiplied the weekly hours by 52 and assumed an average cost per hour of informal help of £7.76. Then, in the same way as we estimated social care costs, we use a two-part regression to estimate informal care use for each age group/gender/IMPACT BAM health state. Specifically, we use a probit for the presence of informal care use, and OLS for the quantity of informal care use for people who use it. The total costs of informal care using these estimates amount to around £28.2bn per year in 2019 prices. The ONS estimated gross value added (GVA) of informal adult care in the UK in 2016 was £59.45bn in 2019 prices. Pro rata-ing this very crudely based on the population of England and Wales being around 88% of UK population, this would be around £55.3billion(5). This is care for all adults aged 18 and over, whereas IMPACT BAM is for population age 35 and over and is mainly accounting for age-related disability.

Overall this method produced a table of informal care costs per year by gender, single year of age (35-100), and IMPACT BAM health state which can then be used as a baseline in the model. The informal care costs are higher for older people and people with dementia which is as would be expected based on other evidence (Figure 6). The informal care costs show an inflection point at around age 60.

Table 9. Data used for estimated cost per hour of informal care. All data from ONS.

| Total people receiving care (2014) | Average hours per recipient per year | Hours per day for people in receipt of care | GVA £bn (2016) | cost per hour | cost per hour 2019£ |
| --- | --- | --- | --- | --- | --- |
| 2,100,000 | 3,857 | 10.57 | 59.45 | £7.34 | £7.76 |

Figure 6. Annual estimated informal care costs by age and health state - IMPACT BAM (average of males & females)

Other possible methods for estimating informal (unpaid) care

We considered using Health Survey for England data or data from a study that used Health Outcomes Data Repository (HODAR) data to estimate informal care from EQ-5D and other characteristics,(6) but decided that using ELSA data would have the best internal consistency with other data we were using.

Table 10. Informal Care in ELSA

| State | No. Obs. |  | Any Informal Help (%) |  | Average Weekly Hours of Informal Help |  | Average Annual Cost (£) |
| --- | --- | --- | --- | --- | --- | --- | --- |
| Free of All | 6736 |  | 4.39% |  | 0.37 |  | 149 |
| CVD | 781 |  | 12.16% |  | 1.64 |  | 662 |
| CVD+CI | 104 |  | 30.77% |  | 4.33 |  | 1,747 |
| CI | 358 |  | 15.92% |  | 2.56 |  | 1,033 |
| CVD+FI | 383 |  | 62.40% |  | 16.47 |  | 6,646 |
| CVD+CI+FI | 145 |  | 58.62% |  | 28.13 |  | 11,351 |
| CI+FI (Dementia) | 186 |  | 59.14% |  | 19.44 |  | 7,844 |
| FI without CI or CVD | 972 |  | 50.21% |  | 10.50 |  | 4,237 |
|  |  |  |  |  |  |  |  |
| Overall Average | -- |  | 14.51% |  | 3.04 |  | 1,227 |

Notes: Data from ELSA waves 6 and 7. Average annual cost is calculated by multiplying average weekly hours by 52 and an assumed average hourly cost of informal care of £7.76.

# 2 Health State Utility Values for Calculating QALYs

QALYs are calculated for individuals in the model as life years * utility index score. In this case, we wanted to use Euroqol 5 dimension 3 level (EQ-5D-3L) which is the UK standard. We searched the CEA registry, the HERC database of mapping studies, and known papers for utility estimates for CVD, cognitive and functional impairment, ADLs, and dementia. For disease-free utility we used UK reference utility values from Janssen & Szende (2014)(7) . One challenge was that the health states of functional and cognitive impairment alone do not correspond directly to specific diseases. Most costs and utility values used in the research literature are measured for specific diseases, typically in cost of illness, burden of disease or pharmaco-economic studies. Given that high costs and low quality of life typically go together, it makes sense that the quality of life decrements for functional impairment are greater than cognitive impairment.

For CVD, dementia and cognitive impairment, the UK reference utility values by age groups were multiplied by values for disease states from the UK EQ-5D MEPS study by Sullivan et al (2011)(8). The EQ-5D MEPS (Medical Expenditure Panel Survey) dataset is often used for health state utility values for models. This is actually based on data from a US population but has a large sample size, controls for comorbidities, and has been crosswalked to UK EQ-5D preference values. For CVD we have used a weighted average of utility multipliers for CHD (ICD 9 codes 410-414, value = -0.0679) and stroke (which itself is an unweighted average of ICD 9 codes 433 precerebral occlusion, 435 transient cerebral ischemia, 436 CVA, 437 other cerebrovascular disease, 438 late effects cerebrovascular disease; overall value = -0.058). For CVD there was an overall value of -0.0613236, based on ratio of prevalence of 3.2 cases of CHD for every 1.7 cases of stroke in England (PHE GP profiles, data for 2016/17)(9). This produced a utility multiplier of (1 – 0.0613) which was 0.93867642.

For each health state we also estimated the utility loss from ADLs based on the distribution of number of ADL deficits (0-6). The utility decrements were drawn from Health Survey for England data from 2012 which included EQ-5D as well as ADLs for people aged 65 and over (N=1,548). HSfE 2012 was the most recent survey that included ADLs and EQ-5D by single year of age. Based on a linear regression of data from this which included age, gender, general health and number of ADLs out of 6, the coefficient for number of ADLs was -0.042. So the utility decrement we used for each ADL was 0.042.

Thus, for each health state/age combination, we multiply the population norm by an EQ-5D multiplier for cognitive impairment or dementia, CVD by the ADL decrements for the distribution of number of ADLs in that state.

Table 11. EQ-5D Utility index values, population norms from Janssen & Szende 2014.

| Population Norms | |
| --- | --- |
|  |  |
| Age Group | UK-England |
|  |  |
| 18–24 | 0.922 |
| 25–34 | 0.914 |
| 35–44 | 0.888 |
| 45–54 | 0.854 |
| 55–64 | 0.814 |
| 65–74 | 0.775 |
| 75+ | 0.706 |
|  |  |
| (All ages 18+) | 0.853 |

Table 12. Utility multiplier values used in the model.

| Health State in Model | ICD 9 code | Decrement | Multiplier |
| --- | --- | --- | --- |
| Dementia | 331 Other cerebral degenerations (which includes Alzheimers) | 0.2165659 | 0.7834341 |
| Cognitive impairment | 294 Persistent mental disorders due to conditions classified elsewhere | 0.0679451 | 0.9320549 |
| CVD | Based on EQ-5D MEPS data applied in ratio of prevalence of 3.2 cases of CHD for every 1.7 cases of stroke in England (PHE GP profiles, data for 2016/17) | 0.06132358 | 0.93867642 |
| Functional impairment | Based on age and number of ADL deficits out of 6, with each ADL producing a decrement of 0.042. | 0.0422411 per ADL | 0.957758873 for 1 ADL |

Table 13. IMPACT BAM states with utility multipliers.

| IMPACT BAM State | Overall utility multiplier  (multiply by EQ-5D by age value) |
| --- | --- |
| Free of all | 1.0000 |
| CVD | 0.9387 |
| CVD+CI | 0.8749 |
| CI | 0.9321 |
| CVD+FI | 0.8506 |
| CVD+CI+FI (CVD+Dementia) | 0.6398 |
| CI+FI (Dementia) | 0.6996 |
| FI without CI or CVD | 0.9181 |

Figure 7. IMPACT BAM, health state utility values by age and model state

## References – Costs and Utility Estimates

1. Licchetta M, Stelmach M. Fiscal sustainability and public spending on health. :45.

2. Health care costs in the English NHS: reference tables for average annual NHS spend by age, sex and deprivation group | Unit Costs of Health and Social Care article database [Internet]. [cited 2019 Oct 24]. Available from: https://www.pssru.ac.uk/ucarticles/2018/01/23/health-care-costs-in-the-english-nhs-reference-tables-for-average-annual-nhs-spend-by-age-sex-and-deprivation-group/

3. Prescription Cost Analysis - England, 2012 [Internet]. NHS Digital. [cited 2019 Oct 24]. Available from: https://digital.nhs.uk/data-and-information/publications/statistical/prescription-cost-analysis/prescription-cost-analysis-england-2012

4. Household satellite account, UK - Office for National Statistics [Internet]. [cited 2019 Oct 4]. Available from: https://www.ons.gov.uk/economy/nationalaccounts/satelliteaccounts/articles/householdsatelliteaccounts/2015and2016estimates

5. Unpaid carers provide social care worth £57 billion - Office for National Statistics [Internet]. [cited 2019 Oct 24]. Available from: https://www.ons.gov.uk/peoplepopulationandcommunity/healthandsocialcare/healthandlifeexpectancies/articles/unpaidcarersprovidesocialcareworth57billion/2017-07-10

6. Rowen D, Dixon S, Hernández-Alava M, Mukuria C. Estimating informal care inputs associated with EQ-5D for use in economic evaluation. Eur J Health Econ. 2016 Jul 1;17(6):733–44.

7. Janssen B, Szende A. Population Norms for the EQ-5D. In: Self-Reported Population Health: An International Perspective based on EQ-5D [Internet]. Springer, Dordrecht; 2014 [cited 2018 Jun 6]. p. 19–30. Available from: https://link.springer.com/chapter/10.1007/978-94-007-7596-1_3

8. Sullivan PW, Slejko JF, Sculpher MJ, Ghushchyan V. Catalogue of EQ-5D Scores for the United Kingdom. Med Decis Making. 2011 Nov 1;31(6):800–4.

9. Public Health Profiles [Internet]. [cited 2019 Oct 24]. Available from: https://fingertips.phe.org.uk/profile/general-practice

###

### Appendix 3. Sensitivity analyses varying value of QALY and discount rates.

**Table 3. Version 2 (sensitivity analysis) – undiscounted costs and QALYs, QALY valued at £30K**

Table 3. Total cumulative undiscounted health and social care costs, value of informal care, and value of QALYs (where 1 QALY valued at £60,000) for adults aged 35-100 in England and Wales, over 10 years from 2020-2029. £billions in 2019 prices (95% uncertainty intervals in brackets). Comparing Scenario 1 – CVD Plateau, with Scenario 2- CVD Fall.

| **Population** | **Scenario** | **Healthcare** | **Social care** | **Value of informal care** | **Total costs** | **Value of QALYs lost (billions)** |
| --- | --- | --- | --- | --- | --- | --- |
|  |  |  |  |  |  |  |
|  | Scenario 1 | 63.0 (49.4 to 75.3) | 12.1 (9.5 to 14.4) | 38.3 (30.1 to 45.8) | 113.4 (89.0 to 135.1) | 38.6 (37.8 to 39.8) |
| **CVD** | Scenario 2 | 49.8 (39.0 to 59.5) | 10.0 (7.8 to 11.9) | 30.9 (24.2 to 36.8) | 90.7 (70.9 to 107.9) | 30.6 (29.8 to 31.7) |
|  | Difference (1-2) | 13.1 (9.8 to 16.8) | 2.1 (1.6 to 2.6) | 7.5 (5.6 to 9.6) | 22.7 (17.0 to 29.1) | 8.1 (9.4 to 6.7) |
|  |  |  |  |  |  |  |
|  | Scenario 1 | 17.4 (13.7 to 21.0) | 52.5 (41.3 to 62.9) | 36.0 (28.2 to 43.2) | 106.0 (83.0 to 127.1) | 21.4 (20.2 to 22.7) |
| **Dementia** | Scenario 2 | 17.6 (13.8 to 21.3) | 52.5 (41.0 to 63.0) | 36.1 (28.1 to 43.5) | 106.2 (82.7 to 127.7) | 21.6 (20.3 to 22.8) |
|  | Difference (1-2) | -0.2 (-1.7 to 1.2) | 0.1 (-2.6 to 2.7) | -0.1 (-2.9 to 2.6) | -0.2 (-7.1 to 6.6) | -0.1 (1.7 to -1.9) |
|  |  |  |  |  |  |  |
|  | Scenario 1 | 800.6 (629.1 to 953.7) | 141.9 (111.9 to 169.4) | 285.2 (224.8 to 339.9) | 1,227.2 (964.8 to 1,460.8) | -7,624.0 (-7,635.5 to -7,610.4) |
| **Whole population aged 35-100** | Scenario 2 | 787.8 (619.4 to 939.0) | 140.5 (110.5 to 167.9) | 277.7 (218.4 to 330.8) | 1,205.5 (948.3 to 1,439.1) | -7,640.0 (-7,651.9 to -7,628.0) |
|  | Difference (1-2) | 12.6 (8.8 to 16.7) | 1.5 (-0.9 to 4.0) | 7.7 (3.4 to 12.8) | 21.6 (13.0 to 32.2) | 16.1 (33.8 to 0.1) |

Note: QALYs are quality adjusted life years. QALYs for CVD and dementia are QALYs lost through disease; QALYs across the whole population is QALYs experienced, so is displayed as a negative value, as it is QALYs lived rather than lost. Data sources same as Table 1.

**Table 3. Version 3 (sensitivity analysis) – costs discounted at 3.5% annually, and QALYs discounted at 1.5%, annually; QALYs, QALY valued at £60K**

Table 3. Total cumulative undiscounted health and social care costs, value of informal care, and value of QALYs (where 1 QALY valued at £60,000) for adults aged 35-100 in England and Wales, over 10 years from 2020-2029. £billions in 2019 prices (95% uncertainty intervals in brackets). Comparing Scenario 1 – CVD Plateau, with Scenario 2- CVD Fall.

| **Population** | **Scenario** | **Healthcare** | **Social care** | **Value of informal care** | **Total costs** | **Value of QALYs lost (billions)** |
| --- | --- | --- | --- | --- | --- | --- |
|  |  |  |  |  |  |  |
|  | Scenario 1 | 52.2 (41.0 to 62.4) | 10.0 (7.8 to 11.9) | 31.8 (25.0 to 37.9) | 94.0 (73.8 to 112.0) | 71.2 (69.6 to 73.3) |
| **CVD** | Scenario 2 | 41.6 (32.5 to 49.7) | 8.3 (6.5 to 9.9) | 25.8 (20.2 to 30.7) | 75.7 (59.2 to 90.1) | 56.5 (55.0 to 58.6) |
|  | Difference (1-2) | 10.6 (7.9 to 13.7) | 1.7 (1.3 to 2.1) | 6.0 (4.4 to 7.7) | 18.3 (13.6 to 23.5) | 14.7 (17.2 to 12.1) |
|  |  |  |  |  |  |  |
|  | Scenario 1 | 14.5 (11.4 to 17.4) | 43.6 (34.3 to 52.2) | 29.9 (23.4 to 35.9) | 88.0 (68.9 to 105.5) | 39.4 (37.2 to 41.9) |
| **Dementia** | Scenario 2 | 14.6 (11.4 to 17.7) | 43.5 (34.0 to 52.3) | 29.9 (23.3 to 36.1) | 88.1 (68.6 to 106.0) | 39.8 (37.5 to 42.0) |
|  | Difference (1-2) | -0.2 (-1.4 to 1.0) | 0.1 (-2.2 to 2.2) | -0.1 (-2.4 to 2.2) | -0.2 (-5.8 to 5.5) | -0.3 (3.2 to -3.5) |
|  |  |  |  |  |  |  |
|  | Scenario 1 | 664.1 (521.9 to 791.2) | 117.5 (92.7 to 140.3) | 236.7 (186.5 to 282.0) | 1,018.0 (800.3 to 1,211.8) | -14,051.2 (-14,072.4 to -14,026.3) |
| **Whole population aged 35-100** | Scenario 2 | 653.8 (514.0 to 779.3) | 116.4 (91.5 to 139.2) | 230.6 (181.4 to 274.7) | 1,000.5 (787.0 to 1,194.3) | -14,080.3 (-14,102.1 to -14,058.3) |
|  | Difference (1-2) | 10.1 (7.0 to 13.5) | 1.2 (-0.8 to 3.2) | 6.2 (2.6 to 10.4) | 17.5 (10.3 to 26.2) | 29.3 (61.7 to -0.2) |

Note: QALYs are quality adjusted life years. QALYs for CVD and dementia are QALYs lost through disease; QALYs across the whole population is QALYs experienced, so is displayed as a negative value, as it is QALYs lived rather than lost. Data sources same as Table 1.

**Table 3. Version 4 (sensitivity analysis) – costs discounted at 3.5% annually, and QALYs discounted at 1.5%, annually; QALYs, QALY valued at £30K**

Table 3. Total cumulative undiscounted health and social care costs, value of informal care, and value of QALYs (where 1 QALY valued at £60,000) for adults aged 35-100 in England and Wales, over 10 years from 2020-2029. £billions in 2019 prices (95% uncertainty intervals in brackets). Comparing Scenario 1 – CVD Plateau, with Scenario 2- CVD Fall.

| **Population** | **Scenario** | **Healthcare** | **Social care** | **Value of informal care** | **Total costs** | **Value of QALYs lost (billions)** |
| --- | --- | --- | --- | --- | --- | --- |
|  |  |  |  |  |  |  |
|  | Scenario 1 | 52.2 (41.0 to 62.4) | 10.0 (7.8 to 11.9) | 31.8 (25.0 to 37.9) | 94.0 (73.8 to 112.0) | 35.6 (34.8 to 36.6) |
| **CVD** | Scenario 2 | 41.6 (32.5 to 49.7) | 8.3 (6.5 to 9.9) | 25.8 (20.2 to 30.7) | 75.7 (59.2 to 90.1) | 28.2 (27.5 to 29.3) |
|  | Difference (1-2) | 10.6 (7.9 to 13.7) | 1.7 (1.3 to 2.1) | 6.0 (4.4 to 7.7) | 18.3 (13.6 to 23.5) | 7.3 (8.6 to 6.1) |
|  |  |  |  |  |  |  |
|  | Scenario 1 | 14.5 (11.4 to 17.4) | 43.6 (34.3 to 52.2) | 29.9 (23.4 to 35.9) | 88.0 (68.9 to 105.5) | 19.7 (18.6 to 20.9) |
| **Dementia** | Scenario 2 | 14.6 (11.4 to 17.7) | 43.5 (34.0 to 52.3) | 29.9 (23.3 to 36.1) | 88.1 (68.6 to 106.0) | 19.9 (18.7 to 21.0) |
|  | Difference (1-2) | -0.2 (-1.4 to 1.0) | 0.1 (-2.2 to 2.2) | -0.1 (-2.4 to 2.2) | -0.2 (-5.8 to 5.5) | -0.1 (1.6 to -1.7) |
|  |  |  |  |  |  |  |
|  | Scenario 1 | 664.1 (521.9 to 791.2) | 117.5 (92.7 to 140.3) | 236.7 (186.5 to 282.0) | 1,018.0 (800.3 to 1,211.8) | -7,025.6 (-7,036.2 to -7,013.2) |
| **Whole population aged 35-100** | Scenario 2 | 653.8 (514.0 to 779.3) | 116.4 (91.5 to 139.2) | 230.6 (181.4 to 274.7) | 1,000.5 (787.0 to 1,194.3) | -7,040.2 (-7,051.1 to -7,029.1) |
|  | Difference (1-2) | 10.1 (7.0 to 13.5) | 1.2 (-0.8 to 3.2) | 6.2 (2.6 to 10.4) | 17.5 (10.3 to 26.2) | 14.7 (30.8 to -0.1) |

Note: QALYs are quality adjusted life years. QALYs for CVD and dementia are QALYs lost through disease; QALYs across the whole population is QALYs experienced, so is displayed as a negative value, as it is QALYs lived rather than lost. Data sources same as Table 1.
