# Supplementary material for: What will the cardiovascular disease slowdown cost? Modelling the impact of CVD trends on dementia, disability, and economic costs in England and Wales from 2020–2029
Source: PLoS One. 2022 Jun 29;17(6):e0268766. doi: 10.1371/journal.pone.0268766 (PMC9242440; doi:10.1371/journal.pone.0268766)
Supplement: Attachment — Submitted filename: IMPACT BAM PLOS One response to reviewers sc (1).docx [file pone.0268766.s010.docx]

**Response to reviewers**

Thank you for submitting your manuscript to PLOS ONE. After careful consideration, we feel that it has merit but does not fully meet PLOS ONE’s publication criteria as it currently stands. Therefore, we invite you to submit a revised version of the manuscript that addresses the points raised during the review process.

To better appreciate your interesting analysis, could you please particularly pay attention to the following excellent points raised by the two reviewers:

1) Please include few paragraphs in the methods section that summarize with sufficient amount of detail how probabilities were defined for transitioning across health states. It is well understood the model simulates an open cohort aged 35-100 over 10 year time horizon. However it is unclear to the reader how new cohorts enter the model (those that turn 35, and would this be on an annual basis), what the initial distributions were for each health state, and how transitions were modelled from disease-free states to disease/death states and from disease to death states (e.g., were transitions based on age and other demographic variables)?

**Thank you. We have added some paragraphs to describe this (p.4) and have added a more description in the Appendix detailing how transition probabilities were calculated.**

**We have also added a new Table 1 with model inputs, as requested below.**

**We have also attached a file with full model inputs. Model inputs vary by single year of age and calendar year so would be very bulky to include in the main body of the paper.**

**We have added more clarity around the new cohort of 35 year olds: A new cohort of those reaching age 35 each year enters through the disease free state as displayed in the arrow in the Model figures. Prevalence of cardiovascular disease and functional impairment is very low in this cohort (<2% in total). Therefore, the resulting error in misclassification is negligible.**

2) Please include a Table 1 summarizing the model parameters and distributions used for transition probabilities, costs and utility weights together with data sources. When a regression equation was used, could you please state this in the table and refer to its source?

**Thank you. We have added a file with all of the model inputs. We have added a new Table 1. Most of the regression equations are quite complex (e.g. lots of age groups); however, all are now included in the Appendix.**

3) Please justify the beta distribution type and OLS for modeling costs. Describe also here how uncertainty was modelled within PSA. Which parameters contributed to the uncertainty and which were assumed to be fixed? How was correlation between parameters incorporated?

**Thank you. The costs were modelled with a two stage least squares regression for the relationship between cost, health state, gender and age. The overall costs were fitted to a beta around +/- 20% to account for other uncertainties that were not accounted for. We used beta based on this paper** [**https://www.york.ac.uk/media/economics/documents/herc/wp/11_31.pdf**](https://www.york.ac.uk/media/economics/documents/herc/wp/11_31.pdf) **- and to account for healthcare costs typically being right-skewed in shape.**

4) Please include more details about the 2 modelled scenarios as indicated by Reviewer 1 and what the implications of assumptions are in the model. Also please clarify whether and which costs depend on gains in life expectancy.

**Thank you. We have added more detail of the 2 modelled scenarios and justification for these scenarios.**

5) Better justify use/non-use of discounting, time horizon and monetary WTP value for a QALY.

**Have added discounted results (using 3.5% for costs and 1.5% QALYs in line with UK Treasury) and £30k valuation of QALYs in Appendix 3.**

6) Please add one-way sensitivity analyses of key parameters as suggested by Reviewer 2.

**We agree that one-way sensitivity analyses, often shown in the form of tornado charts are really useful in showing the key sources of uncertainty around incremental net monetary benefit between two or more interventions. However, this paper is not a traditional cost effectiveness analysis comparing an intervention with a comparator. It is comparing two scenarios where the sources of uncertainty are generally the same for both scenarios so would cancel each other out somewhat in a tornado diagram. We are planning to carry out more work in the future looking at the drivers in healthcare costs in different model scenarios using blinder-oaxaca decomposition but we feel it is beyond the scope of the present paper which is quite lengthy already.**

In addition, several more textual suggestions were made by both reviewers.
Please submit your revised manuscript by Dec 20 2021 11:59PM. If you will need more time than this to complete your revisions, please reply to this message or contact the journal office at [plosone@plos.org](mailto:plosone@plos.org). When you're ready to submit your revision, log on to <https://www.editorialmanager.com/pone/> and select the 'Submissions Needing Revision' folder to locate your manuscript file.

Please include the following items when submitting your revised manuscript:

- A rebuttal letter that responds to each point raised by the academic editor and reviewer(s). You should upload this letter as a separate file labeled 'Response to Reviewers'.
- A marked-up copy of your manuscript that highlights changes made to the original version. You should upload this as a separate file labeled 'Revised Manuscript with Track Changes'.
- An unmarked version of your revised paper without tracked changes. You should upload this as a separate file labeled 'Manuscript'.

If you would like to make changes to your financial disclosure, please include your updated statement in your cover letter. Guidelines for resubmitting your figure files are available below the reviewer comments at the end of this letter.

If applicable, we recommend that you deposit your laboratory protocols in [protocols.io](http://protocols.io/) to enhance the reproducibility of your results. Protocols.io assigns your protocol its own identifier (DOI) so that it can be cited independently in the future. For instructions see: <https://journals.plos.org/plosone/s/submission-guidelines#loc-laboratory-protocols>. Additionally, PLOS ONE offers an option for publishing peer-reviewed Lab Protocol articles, which describe protocols hosted on [protocols.io](http://protocols.io/). Read more information on sharing protocols at <https://plos.org/protocols?utm_medium=editorial-email&utm_source=authorletters&utm_campaign=protocols>.

We look forward to receiving your revised manuscript.

Kind regards,

Bart Ferket
Academic Editor
PLOS ONE

Journal Requirements:

When submitting your revision, we need you to address these additional requirements.

1. Please ensure that your manuscript meets PLOS ONE's style requirements, including those for file naming. The PLOS ONE style templates can be found at

<https://journals.plos.org/plosone/s/file?id=wjVg/PLOSOne_formatting_sample_main_body.pdf> and <https://journals.plos.org/plosone/s/file?id=ba62/PLOSOne_formatting_sample_title_authors_affiliations.pdf>

2. Please amend the manuscript submission data (via Edit Submission) to include authors: Piotr Bandosz, Maria Guzman-Castillo, Jonathan Pearson-Stuttard, George Stoye, Jeremy McCauley, Sara Ahmadi-Abhari, Marzieh Araghi, Martin J Shipley, Simon Capewell, Eric French, Eric J Brunne, Martin O’Flaherty

3. Thank you for stating the following in the Competing Interests/Financial Disclosure section:

“All authors have completed the Unified Competing Interest form (available on request from the corresponding author) and declare: no support from any organisation for the submitted work; no financial relationships with any organisations that might have an interest in the submitted work in the previous three years. Dr Collins is secondment as Head of Health Economics in Welsh Government, this paper does not represent any views of Welsh Government. Dr Pearson-Stuttard is also Head of Health Analytics at Lane Clark & Peacock LLP, vice-chair of the Royal Society for Public Health and reports personal fees from Novo Nordisk A/S, all outside of the submitted work.”

We note that one or more of the authors are employed by a commercial company:Health Analytics at Lane Clark & Peacock LLP

a. Please provide an amended Funding Statement declaring this commercial affiliation, as well as a statement regarding the Role of Funders in your study. If the funding organization did not play a role in the study design, data collection and analysis, decision to publish, or preparation of the manuscript and only provided financial support in the form of authors' salaries and/or research materials, please review your statements relating to the author contributions, and ensure you have specifically and accurately indicated the role(s) that these authors had in your study. You can update author roles in the Author Contributions section of the online submission form.

Please also include the following statement within your amended Funding Statement.

“The funder provided support in the form of salaries for authors [insert relevant initials], but did not have any additional role in the study design, data collection and analysis, decision to publish, or preparation of the manuscript. The specific roles of these authors are articulated in the ‘author contributions’ section.”

**Thanks have added this about the funder BHF.**

If your commercial affiliation did play a role in your study, please state and explain this role within your updated Funding Statement.

b. Please also provide an updated Competing Interests Statement declaring this commercial affiliation along with any other relevant declarations relating to employment, consultancy, patents, products in development, or marketed products, etc.

Within your Competing Interests Statement, please confirm that this commercial affiliation does not alter your adherence to all PLOS ONE policies on sharing data and materials by including the following statement: "This does not alter our adherence to  PLOS ONE policies on sharing data and materials.” (as detailed online in our guide for authors <http://journals.plos.org/plosone/s/competing-interests>) . If this adherence statement is not accurate and  there are restrictions on sharing of data and/or materials, please state these. Please note that we cannot proceed with consideration of your article until this information has been declared.

Please include both an updated Funding Statement and Competing Interests Statement in your cover letter. We will change the online submission form on your behalf.

**Thank you. We have now added this.**

[Note: HTML markup is below. Please do not edit.]

Reviewers' comments:

Reviewer's Responses to Questions

**Comments to the Author**

1. Is the manuscript technically sound, and do the data support the conclusions?

The manuscript must describe a technically sound piece of scientific research with data that supports the conclusions. Experiments must have been conducted rigorously, with appropriate controls, replication, and sample sizes. The conclusions must be drawn appropriately based on the data presented.

Reviewer #1: Partly

Reviewer #2: Yes

2. Has the statistical analysis been performed appropriately and rigorously?

Reviewer #1: Yes

Reviewer #2: Yes

3. Have the authors made all data underlying the findings in their manuscript fully available?

The [PLOS Data policy](http://www.plosone.org/static/policies.action#sharing) requires authors to make all data underlying the findings described in their manuscript fully available without restriction, with rare exception (please refer to the Data Availability Statement in the manuscript PDF file). The data should be provided as part of the manuscript or its supporting information, or deposited to a public repository. For example, in addition to summary statistics, the data points behind means, medians and variance measures should be available. If there are restrictions on publicly sharing data—e.g. participant privacy or use of data from a third party—those must be specified.

**We have included a file with a detailed set of model inputs. We plan to publish the complete model at some point in the future, but need to work through it with funders and authors.**

Reviewer #1: No

Reviewer #2: No

4. Is the manuscript presented in an intelligible fashion and written in standard English?

PLOS ONE does not copyedit accepted manuscripts, so the language in submitted articles must be clear, correct, and unambiguous. Any typographical or grammatical errors should be corrected at revision, so please note any specific errors here.

Reviewer #1: No

Reviewer #2: Yes

5. Review Comments to the Author

Please use the space provided to explain your answers to the questions above. You may also include additional comments for the author, including concerns about dual publication, research ethics, or publication ethics. (Please upload your review as an attachment if it exceeds 20,000 characters)

Reviewer #1: This study is a model-based analysis of the economic implications of the recent trend in CVD incidence in England and Wales compared to a counterfactual of persisting long-term trend. Using a probabilistic Markov model (IMPACT-BAM), the study simulated and compared two scenarios: 1) CVD Plateau, where CVD incidence remains at 2011 levels, and 2) CVD Fall, where CVD incidence follows the long-term trends and continues to decline. It concludes that the slowdown in the decline of CVD incidence is associated with substantial costs.

The topic is potentially interesting, and the findings could help guide priority setting in the local setting. Unfortunately, key methodological details were not sufficiently described in the text to determine the validity of the study. I have included some major issues mostly around the methods and other comments and questions in the following section.

Major issues:
1. The methods section lacks essential details. I understand that the study used a previously published simulation model, but it was unclear how the two scenarios were implemented exactly in this particular study. For example, how was the trend of CVD incidence in the two scenarios fitted statistically? What is the risk profile of those who develop CVD in scenario 1 compared to scenario 2? Considering that the comparison between these two scenarios is the central question of this study, it was very difficult to determine its methodological validity.

**Thank you, we have added more detail for the methods and for the two scenarios to the paper and the appendix.**

2. It was also unclear how CVD and dementia were correlated in this analysis, i.e., how a change in CVD incidence trend between two scenarios affects the health and cost outcomes of dementia, and how this effect was operationalized in the simulation model.

**Thank you. The model is built on ELSA data where having CVD increases the probability of transition to dementia vs. a no dementia state. We have included more detail of this in the paper and appendix.**

3. A decrease in CVD incidence is associated with longer life expectancy, but this prolonged life expectancy also comes with some future unrelated healthcare costs: if we prevent individuals from developing CVD, they may still seek care and incur health spending because of other conditions. Not accounting for such costs may bias the results in favor of the CVD Fall scenario. I could not find whether (and if yes, how) this study includes such costs from the description of the methods.

**Thank you. The study includes future unrelated healthcare costs. We have made this more clear in the description. This is one of the key questions we aim to answer: does a CVD reduction save money and QALYs even if it means people live longer. The answer suggested by our study is yes.**

4. Since this study projects into the next 10 years to calculate the economic impact of CVD trends, one would think that discounting is crucial to account for time preferences. However, it was not clear why discounting was not performed, and I think it would help to see both discounted and undiscounted results.

**Thank you. We have added a discounted sensitivity analysis – at 3.5% for costs, 1.5% for QALYs, , in line with NICE and UK Treasury. Discounting was not initially carried out because we are not looking at a specific intervention and wanted to give an idea of how health system costs will evolve year on year, which discounting may partially obscure.**

5. The manuscript requires significant copyediting. References are sometimes misaligned (e.g., Introduction -> paragraph 3 -> line 3, the ELSA study is referenced to #8, but should be #11 instead). Many sections/paragraphs lack structure and focus -- the “Implications for policy makers” section is a prime example consisting of scattered paragraphs of just 1-2 sentences; they should be combined to indicate a clear flow of logic. Another example: the “Social care and informal care costs” section includes some methodological descriptions of the QALYs, which should belong to the next section. Some abbreviations are used before they are defined (e.g., “HES”). Additionally, there are grammar errors (incorrect punctuations and capitalizations, etc.) throughout the manuscript.

**Thank you, we have corrected these examples and have conducted a further round of copy editing.**

Other comments:
1. The study uses £60,000/QALY as the threshold to value QALYs and references the UK Treasury Green Book. However, the most often used threshold we see from the UK is £30,000/QALY recommended by NICE, substantially different from £60,000/QALY. So, what is the rationale for using £60,000/QALY? I also wonder how the results would change if alternative thresholds were used.

**Thank you. We have added a sensitivity analysis using £30k per QALY. The NICE threshold is used for recommending technologies for the NHS which has a (typically) fixed budget. The UK Treasury valuation is used for considering policy interventions which may be more appropriate for the present study.**

2. Why were costs fitted to beta distributions? Beta distributions are more commonly used for probabilities and utility weights in probabilistic sensitivity analysis, and Gamma or Log-normal are more appropriate for costs.

**Thank you. We used beta based on this paper which suggests GB2 can be the best distribution for healthcare costs which are often right-skewed** [**https://www.york.ac.uk/media/economics/documents/herc/wp/11_31.pdf**](https://www.york.ac.uk/media/economics/documents/herc/wp/11_31.pdf)

3. I’m having a hard time understanding what this sentence means: “The QALYs reflect only the uncertainty in the epidemiology, not uncertainty around the QALY impact of disease, which is reflected in very tight confidence intervals.” Is this essentially saying that there’s no probabilistic distribution added to the QALY weights because the uncertainty on QALY weights is very low? In any case, this sentence should be reworded to improve clarity.

**Thank you, yes. We have reworded it as you suggest to make it clearer.**

4. Tables 1 and 2: These tables present results for a “base case scenario.” It’s confusing because a base case scenario is not described and defined in the methods section.

**Thank you for picking this up. We have changed this – it is actually Scenario 2, although the results for this are very similar for both scenarios.**

5. Scenario 2 is a hypothetical scenario where CVD incidence declines at a rate close to historical levels. Even though the discussion section touches briefly on the aspects CVD prevention could focus on, scenario 2 seems like a very hypothetical and unrealistic scenario without some justification on *how* this decline could be achieved. More and deeper discussion on this could be helpful, and maybe a somewhat more realistic scenario could be of more policy interest.

**Thank you. We give some examples of countries that have continued to show reductions in CVD incidence and that have much lower incidence than the UK so we do not believe the scenario we are presenting is unrealistic, however it may take a longer time to achieve, especially given the pandemic.**

6. The “Future research” section claims that “Future research could use our IMPACT-BAM model to look further at health inequalities, and produce more granular estimates at regional or at local authority levels, especially given the increasingly local input to resource allocation.” It was unclear, though, whether the authors have made the IMPACT-BAM model publicly available for such uses. I could not find a user interface for the model from an online search.

**Thank you. We aim to make the IMPACT-BAM model available in due course. Furthermore, we have a big group working on the model, so some future research may be carried out by people with previous experience of working with the model.**

Reviewer #2: This is a well-conducted and important study that quantifies the costs and QALYs associated with persistent plateauing versus continued decline of cardiovascular disease rates in England and Wales.

Below I provide some comments on each section of the abstract. While most of these are minor recommendations, I strongly recommend that the simulation procedure is more clearly explained in the Methods section and that sensitivity analysis is conducted to better quantify the contribution of individual model parameters to overall uncertainty.

Abstract
The abstract provides a detailed description of the study.

Introduction
The Introduction details declining CVD mortality rates during the late twentieth century and their subsequent plateauing. The respective causes of declines in CVD and CHD mortality in the U.K. have been discussed in prior literature (e.g., Bajekal et al., PLoS Med, 2012 and O’Flaherty, Buchan, and Capewell, Heart, 2012). Some discussion of the relative impact of different risk factor exposures and novel treatments would be informative.

**Thank you. We have added some reference to treatments that may reduce the CVD case fatality ratio; we are mainly concerned with CVD incidence in this study related to policy interventions, rather than tertiary prevention but agree tertiary prevention is important.**

The Introduction clearly sets out the important relationship between CVD and dementia, explains the need for a study to estimate future health and cost consequences associated with the ‘cardiovascular disease slowdown’, and summarises how this will be achieved.

Methods
The methods are well-described and are appropriate for the research question, employing a previously validated Markov model. Previous publications which employed the model should be cited when the IMPACT BAM model is introduced in the Methods section. Further information on the basis for the U.K. treasury’s decision to value QALYs at £60,000 may provide context to this model parameter (i.e., determined based on revealed preference studies which aim to quantify the statistical value of a life). A short justification for the study time horizon would also be helpful.

**Thank you. We have added reference to the study time horizon mirroring the NHS plan. We have also added sensitivity analysis with £30k / QALY valuation.**

Generally, the Methods section would be improved with a subsection which clearly describes the simulation approach. The manuscript refers to ‘probabilistic sensitivity analysis’ once in the Methods and once in the Strengths and Limitations sections. It would be useful to have a short section of the Methods which describes how cohorts transition through the model (including a model figure, as recommended by Consolidated Health Economic Evaluation Reporting Standards guideline), how many cycles are included in each run, the process by which parameters are stochastically sampled, the approach to defining probability distributions for each of the model parameters, and the way summary statistics from probabilistic runs are reported. I would strongly advise inclusion of a ‘Table 1’ which describes all the model inputs, and their mean values, ranges, distributions for probabilistic analysis, and sources. The suggested figure and table could be included in the supplement material.

**Thank you. We have added a Table 1, and model figure into the Appendix.**

The probabilistic simulation approach helps to quantify the uncertainty inherent in this modelling study. The contribution of individual model parameters to this uncertainty is unclear. I recommend conducting ‘traditional’ sensitivity analysis, whereby most parameters are held constant while some parameters are systematically varied (either deterministically or probabilistically). The impact of these results on important outcomes (e.g., incremental health, social and care and total costs) could be presented in tornado diagrams.

**Thank you. Please see comment above. We aim to carry out future work looking in more detail at the drivers of change and uncertainty in the model.**

Results
The results section is well-written, containing all relevant results. The number of QALYs and life years accumulated in both scenarios are important intermediate outcomes that could also be reported.

**Thank you. We have made more reference to the incremental QALY results.**

Discussion
The discussion provides a useful summary of the results, sets the manuscript in the context of similar literature, and outlines strengths and limitations. The section ‘Implications for Policy Makers’ may be improved by citing examples of cost-effective cardiovascular prevention policies which could be implemented by policy-makers to arrest the plateauing of CVD rates.

**Thank you. We have added more reference to cost effective prevention policies in this section.**

**Huge thanks to both reviewers and to the editors for providing such perspicacious comments. We hope that we have acted upon them to your satisfaction, and that our paper is now fit for publication in** **PLOS ONE.**
