# Supplementary material for: What will the cardiovascular disease slowdown cost? Modelling the impact of CVD trends on dementia, disability, and economic costs in England and Wales from 2020–2029
Source: PLoS One. 2022 Jun 29;17(6):e0268766. doi: 10.1371/journal.pone.0268766 (PMC9242440; doi:10.1371/journal.pone.0268766)
Supplement: Attachment — Submitted filename: PLOS One response to reviewers 20220427.docx [file pone.0268766.s011.docx]

**Response to Reviewers**

Dear PLOS One

Thank you for reviewing our study again. Please find rebuttal below;

**Please perform the additional one-way sensitivity analyses as suggested or provide a better justification for not performing these in the discussion (strengths and weaknesses) section in response to the remaining concerns of the reviewer. Note that the sentence "Hence the value of our extensive sensitivity analyses." may not be supported well by your analyses in the current version based on the comments of the reviewer.**

Thank you for this comment. We have taken out this sentence and replaced it with sentences below. We have included some one-way sensitivity analyses as suggested by reviewers. We have included probabilistic sensitivity analysis which is generally seen as the gold standard in terms of sensitivity analysis but the way the model is structured makes it more difficult to perform a series of one-way sensitivity analyses, but we agree that this would be useful and it is something we intend to do in future studies with this model, to explore the drivers of change over time. So we agree with the reviewer that this will be useful, but we want to explore it more in a potential future study.

*“We have included some one-way sensitivity analyses (value of QALY, discount rate), as well as the probabilistic sensitivity analysis; but more one-way sensitivity analyses may highlight the drivers of differences in outcomes in more detail. We plan to produce a future study to explore these drivers more, using decomposition analysis.”*

I hope this change in emphasis as well as explaining our future plans answers this to your satisfaction.

With best wishes

Dr Brendan Collins
